# Editorial: Advances in Optics and Acoustics Towards Translational Functional Neuroimaging

**DOI:** 10.3389/fnins.2022.868402

**Published:** 2022-03-23

**Authors:** Jeeun Kang, Jae-Ho Han, Ping Yan

**Affiliations:** ^1^Laboratory for Computational Sensing and Robotics, Johns Hopkins University, Baltimore, MD, United States; ^2^Department of Brain and Cognitive Engineering, Korea University, Seoul, South Korea; ^3^R. D. Berlin Center for Cell Analysis and Modeling, University of Connecticut Health Center, Farmington, CT, United States

**Keywords:** neuroimaging, optical imaging, optical imaging analysis, voltage sensitive dye, membrane potential, acoustic imaging

Functional neuroimaging at high spatiotemporal resolution is an ultimate goal of neuroimaging either to support neuroscientific investigations or to guide clinical procedures in distinct neural systems from the brain to neuromuscular systems, and to peripheral nerve networks.

There are several conventional neuroimaging modalities available in clinics, but they have limitations suggesting desperate needs for new modality. Positron emission tomography (PET) provides stark molecular and pharmacological contrast but suffers from painful temporal resolution at low spatial specificity. Functional magnetic resonance imaging (fMRI) offers a higher spatial resolution of functional neural activity in whole-brain; however, the recorded blood-oxygenation level-dependent (BOLD) signal involves uncertain interpretation at low temporal resolution limited by hemodynamic response time (~10 s). Also, in PET and fMRI, the expansion into interventional guidance has been prevented other than diagnostics, due to no flexibility in system configuration. Conventional optical imaging has offered high temporal resolution, but have limited dynamic ranges and cover only superficial tissue depths, often requiring invasive craniotomy with problematic long-term consequences abandoning translational practicality to non-human primate and to human studies (Kim et al., 2017; Han and Cha, 2020b; Tayebi et al., 2020).

This Research Topic aimed to explore recent advances in optics and acoustics toward translational functional neuroimaging. For the last few decades, optical, acoustic, and photoacoustic neuroimaging modalities have thrived with promising progress, based on their advantages of versatile imaging configuration and spatiotemporal resolution, adaptable exogenous contrast, compactness, and minimal or total non-invasiveness. The Research Topic surveys the field in two primary technological edges.

There were several investigations on testing, validating, and translating new chemical tools. Walker et al. presented a new membrane potential dye, Berkeley Red Sensor of Transmembrane potential-1 (BeRST 1) to enable a monitoring of dozens of neurons at a sampling rate of 500 Hz. The capability of the far-red sensor was tested in cultures of rat hippocampal neurons. High fidelity was found by fluorescence imaging and patch-clamp electrophysiology. They also elaborate a workflow using a semi-automated optical spike and connectivity analysis (OSCA) program preventing any user bias during the research. The OSCA framework presented feasibilities for investigations on acute, pharmacological interventions (e.g., gabazine and picrotoxin) and on a continuous monitoring of chronic perturbations of network connectivity (e.g., exposure to amyloid beta 1-42). Moreover, the OSCA framework was able to capture gradual changes of spiking patterns as the cultures being maturated. In a separate article, the same group deployed the OSCA framework to investigate preferential wiring of excitatory hippocampal neurons using same sensor, BeRST 1. Thanks to its high spatiotemporal resolution, they were able to reconstruct a functional map connecting from an origin neuron to downstream neurons in a spontaneously active network of cultures raised on microislands, parallelizing the developments of inhibitory and excitatory cell types. In their study, the authors found a preferential connectivity between DGC and CA3 neurons and between CA3 and CA1 neurons, encouragingly well mimic the circuitry in an intact hippocampus. They are eager to expand the research to test diverse neuronal cell types and in different neuronal health states.

Kirk et al. proposed a hybrid rhodamine voltage reporter (RhoVR) combined with the HaloTag self-labeling enzyme (RhoVR-Halo) as a *in vivo* imaging tool to track membrane potential changes, based on their successful studies in neuronal cultures and *ex vivo* slices. In the article, authors developed a transgenic reporter line in *Drosophila* where the HaloTag enzyme to be expressed on the cell surface, so that cell type-specific labeling could be achieved. The study suggests a step further advance toward epifluorescence two-photon microscopy at high spatiotemporal resolution.

Pak et al. presented an optimization of near-infrared fluorescence voltage-sensitive dye (VSD) for non-invasive neuronal activity monitoring in rodents. The main goal of the study was to optimize the transmembrane redistribution mechanism of cyanine VSD in different chemokinetic environments in tubing phantom, *in vitro* cultures, *ex vivo* brain slice, and *in vivo* brain. The study is based on their previous study on pharmacological blood-brain barrier (BBB) disruption technique (Pak et al., 2018), and further suggests a specific range of VSD concentration for successful systematic delivery into brain, while avoiding natural VSD aggregation due to high VSD density in the solution and so bloodstream. Authors suggested its clinical translation of the cyanine VSD technology for a functional guidance of nerve-sparing surgeries (Kang et al., 2020b) and multi-modal neuroimaging with photoacoustic imaging with deep-tissue contrast on neural activations reciprocal to fluorescence contrast changes (Zhang et al., 2017; Kang et al., 2019, 2020a; Han and Cha, 2020a).

Other studies investigated bridging from micro- to macro-world observations in neuroscience. Jang et al. presented the combinational observation of whole-cell patch clamping outcome with individual presence of different genetically encoded voltage indicators (GEVIs), in which temporal membrane voltage trace in the target pyramidal neurons can be directly read in a same temporal domain of the population voltage changes in the surrounding neuropil of brain slices. All of four candidate GEVIs (i.e., ArcLightD, chi-VSFP, di-4-ANEPPS, and Archon1) presented acceptable optical imaging performances to characterize the spatiotemporal voltage changes in neuropil, but the authors clarified that slow voltage transient time is a primary source of signal contamination such as indistinguishable neuronal activations and more difficult photobleaching correction. The authors concluded with the benefit of the simultaneous framework of synapse stimulation, whole-neuron recording, and optical imaging.

In relatively wider field-of-view, Cecchetto et al. compared the two-photon imaging of VSD (ANNINE-6plus) or calcium indicator (GCaP6f) with multi-channel local field potential (LFP) recordings. The observation was focused on different layers of the barrel cortex from anesthetized and awake through craniotomy window. What they found was aligned with prior work showing that anesthesia blocks the neuronal mechanisms in pyramidal neurons; sensory stimuli inactivates as well as enhances cortical dynamics; and further produced interesting observations of negative calcium responses in response to vibrissa stimulation in awake mice. The authors introduce a multimodal neuronal investigation approach to general neuroscience community.

In summary, this Research Topic brought together several notable recent advances in non-invasive neuroimaging technologies for scientific and clinical innovations.

## Author Contributions

JK wrote the first draft. JK, J-HH, and PY edited and confirmed the article. All authors contributed to the article and approved the submitted version.

## Funding

This work was supported in part by the National Research Foundation of Korea (NRF) grant funded by the Korean government (MSIT) (NRF-2020R1A4A1018309), in part by the MSIT (Ministry of Science and ICT), Korea, under the ICT Creative Consilience program (IITP-2022-2020-0-01819) supervised by the IITP (Institute for Information and communications Technology Planning and Evaluation), and in part by Louis B. Thalheimer Fund for Translational Research.

## Conflict of Interest

The authors declare that the research was conducted in the absence of any commercial or financial relationships that could be construed as a potential conflict of interest.

## Publisher's Note

All claims expressed in this article are solely those of the authors and do not necessarily represent those of their affiliated organizations, or those of the publisher, the editors and the reviewers. Any product that may be evaluated in this article, or claim that may be made by its manufacturer, is not guaranteed or endorsed by the publisher.
